# Accessibility to Obstetric Care in South Florida Based on Insurance: A Cross-Sectional Study

**DOI:** 10.7759/cureus.44781

**Published:** 2023-09-06

**Authors:** Maria Kolesova, Sydney Sarantos, Juan Alvarez, Alfred Torres, Soniya Pateriya, Manuel Penalver

**Affiliations:** 1 Medical School, Florida International University, Herbert Wertheim College of Medicine, Miami, USA; 2 Obstetrics and Gynecology, Florida International University, Herbert Wertheim College of Medicine, Miami, USA

**Keywords:** cigna, united healthcare, medicaid, prenatal care, obstetrics, healthcare insurance

## Abstract

Introduction

Obstetrical research confirms that earlier onset prenatal care significantly improves pregnancy and birth outcomes. Initiating care in the second trimester or having less than 50% of recommended visits has been associated with an increased risk of prematurity, stillbirth, neonatal, and infant death. Studies have shown that women on public health insurance plans initiate prenatal care substantially later into pregnancy than those on private plans. The purpose of this study is to assess whether public health insurance limits Florida patients’ access to obstetric care.

Methods

A cross-sectional study was conducted by collecting data on the four most populated zip codes for Medicaid in South Florida using HealthGrades.com. The following search parameters were used: “obstetric care”, “four stars and up” and “10-mile distance”. Each obstetrician was called three times to assess appointment availability for fictional nulliparous women at eight weeks of gestation requesting prenatal care. Accepted insurance types (Medicaid, Cigna, and United Health Group (UHG)), time to an appointment in business days, and self-pay rates were recorded. Practices with invalid contact information and retired obstetricians were excluded. Summary statistics, chi-squared analysis, and a two-way t-test were conducted for the primary outcome.

Results

Seventy-one out of 178 obstetricians were successfully contacted, of which 31 physicians accepted all three insurances, and 40 physicians did not accept at least one insurance. Of those, 97.2% accepted UnitedHealthcare, 98.6% accepted Cigna, and 45.1% accepted Medicaid. There was a statistically significant difference when comparing acceptance rates between UHC and Medicaid as well as Cigna and Medicaid (p<0.001). There was no statistically significant difference in acceptance rates in the direct comparison of the two private insurances, Cigna and UnitedHealthcare (p=0.559). The average number of days until the next available appointment was 12.7 (SD= 7.2) for UnitedHealthcare, 20.0 (SD=6.7) for Cigna, and 17.0 (SD=8.6) for Medicaid. There was a statistically significant trend between the type of insurance and the time to the earliest appointment (p=0.002).

Conclusion

This study demonstrated patients enrolled in Medicaid in South Florida have significantly less access to prenatal care than those with private insurance. This evidence shows that decreased access to care from Medicaid plans can possibly increase the risk of adverse outcomes associated with inadequate prenatal care. This information should be considered by policymakers when considering future Medicaid expansion.

## Introduction

Access to prenatal care ensures positive birth outcomes and prevents maternal and infant morbidity and mortality [1]. Unfortunately, insurance status can prevent expectant mothers from receiving adequate prenatal healthcare. The lack of access to prenatal care, notably the delayed initial prenatal appointment, can result in delayed diagnostic testing and genetic developmental screenings, which can have long-term health consequences for the mother and fetus. Furthermore, inadequate prenatal care is associated with an increased risk of stillbirth and neonatal and infant death [1, 2]. 

Unfortunately, previous studies have shown that private insurance programs compared to public health insurance Medicaid were associated with a 3.3-fold higher likelihood of scheduling an appointment when seeking specialty care and a 1.6-fold higher likelihood when seeking primary care [3]. Additionally, the percentage of women with the late beginning of prenatal care among women with public health insurance was consistently higher than those with private insurance [4].

This study aims to investigate whether public health insurance programs limit Florida patients' access to obstetric care compared to private insurance programs. Specifically, we aim to assess the access to prenatal care and wait times for females in South Florida covered by Medicaid, Cigna, and UnitedHealthcare (UHC). We hypothesize that Medicaid, a public health insurance program, will limit South Florida patients' access to prenatal care compared to Cigna and UnitedHealthcare private insurance programs. Our research employs a "secret-shopper" methodology to investigate obstetricians' availability and acceptance rates based on the insurance type in the four most densely populated zip codes concerning Medicaid enrollment in South Florida. This research could contribute to identifying potential solutions to address the disparities in access to obstetric care based on insurance coverage.

## Materials and methods

To conduct this cross-sectional study, FlHealthCharts.gov was used to identify the four Florida county zip codes with the highest amount of Medicaid enrollees. Miami-Dade, Broward, Hillsborough, and Palm Beach County were included. Using these zip codes, HealthGrades.com, a popular website used by patients to find local providers, was queried to identify local obstetricians. In order to replicate a patient's interaction with this website, the following search parameters were used to obtain the contact information for obstetricians local to each zip code: "obstetric care," "four stars and up," and "10-mile distance." Implementing these parameters, HealthGrades produced a list of 178 obstetricians across all four included zip codes. Physicians excluded from the study were those who offered only gynecologic but not obstetric care, were retired, permanently closed, or were no longer accepting new patients. Physicians were also excluded if their originally provided contact information was incorrect and an alternative phone number could not be found via Google search. 

Each doctor's office was then called and queried for three different insurances. The insurance plans chosen were Medicaid, which represents government insurance, and two forms of private insurance with wide coverage across Florida, Cigna and UHC. Each practice was then called three different times, once per insurance type, requesting prenatal care appointment availability for a fictional nulliparous 24-year-old woman currently at eight weeks gestation. To prevent voice recognition, researchers were assigned to make individual calls for each type of insurance. Using this "secret shopper" study design, each researcher would call between 9:00 a.m. and 4:30 p.m. using a premade script to assess appointment availability by insurance type. 

A designed script simulated a patient, or the spouse of a potential patient, looking to establish an initial prenatal appointment after receiving a positive urine pregnancy test. The secret shopper then informed the receptionist that their estimated gestational age was eight weeks, based on their last menstrual cycle. They then inquired about the next available appointment for an initial prenatal visit. During the call, callers noted whether the assigned insurance was accepted by the obstetrician, which was used as the study's primary outcome. If the insurance was accepted, the time to the next appointment, measured in a number of business days, was denoted as the study's secondary outcome. 

Researchers were strictly instructed to refrain from booking or confirming an appointment in order to avoid taking away resources from actual patients [5]. Callers were also instructed on handling scenarios surrounding poor contact with the office, such as being sent directly to voicemail or placed on hold for more than 15 minutes, which were managed by re-calling the office on a different date. If the physician's office could not be reached on three separate occasions, the physician was ultimately excluded from the study due to poor contact. 

In accordance with similar secret shopper studies, this study did not meet the requirements of "human subject" research as only organizational data on insurance acceptance rates and time to appointment were collected through the phone call survey. Therefore, as only organizational data was collected, this study did not require institutional review board approval or informed consent [6, 7]. 

Statistical analysis 

All data were collected on Microsoft Excel Online using Microsoft Forms for data entry. Data analysis was performed with Microsoft Excel 365 (Microsoft Inc., Redmond, WA). Chi-squared analysis was employed to compare associations between qualitative variables. The ANOVA test was used to compare continuous variables. Statistical significance was assigned at an alpha value of 0.05. 

## Results

**Figure 1:**
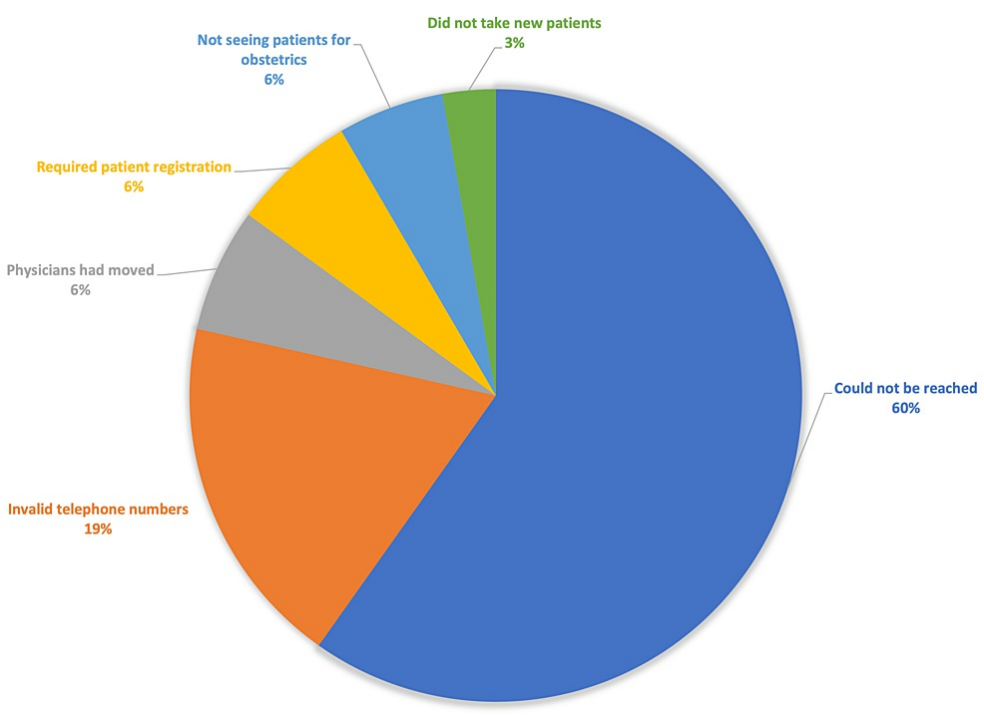
Exclusion Criteria for Obstetricians’ Availability in Study The chart displays the percentage breakdown of reasons why 107 physicians were excluded from the study. The breakdown shows that 60% could not be reached after three attempts, 19% had phone numbers that were invalid, 6% had moved, 6% required patients to register, and 6% did not provide obstetrics services. Only 3% were not accepting new patients.

The four most populated zip codes for Medicaid enrollment in South Florida were Miami-Dade, Broward, Hillsborough, and Palm Beach. The search on Healthgrades used parameters of obstetric care, a rating of four stars or more, and a 10-mile distance range from the given zip code. Using the criteria outlined above on FlHealthCharts.gov, a sample of 178 obstetricians was obtained. Out of the 178 obstetricians sampled, 107 physicians were excluded from the analysis. Out of 107 physicians that were excluded, 64 could not be reached, 20 had invalid telephone numbers, seven had moved to a different location, seven required patient registration before providing information, six reported not seeing patients for obstetrics, and three did not take new patients (Figure 1). Seventy-one physicians were successfully reached for all three insurances and were used for the following data analysis. In this initial analysis, 97.2% accepted UnitedHealthcare, 98.6% accepted Cigna, and 45.1% accepted Medicaid (Table 1). There was a statistically significant difference when comparing acceptance rates between UHC and Medicaid as well as Cigna and Medicaid (p<0.001). There was no statistically significant difference in acceptance rates in the direct comparison of the two private insurances, Cigna and UnitedHealthcare (p=0.559). 

**Table 1:** Total Insurance Acceptance This table includes the total number of obstetricians successfully contacted, as well as their acceptance and rejection of each insurance type, including UnitedHealthcare, Cigna and Medicaid.

Insurance	Number of physicians accepting insurance	Number of physicians not accepting insurance	Total number of physicians contacted
UnitedHealthcare	69	2	71
Cigna	70	1	71
Medicaid	32	39	71

**Figure 2:**
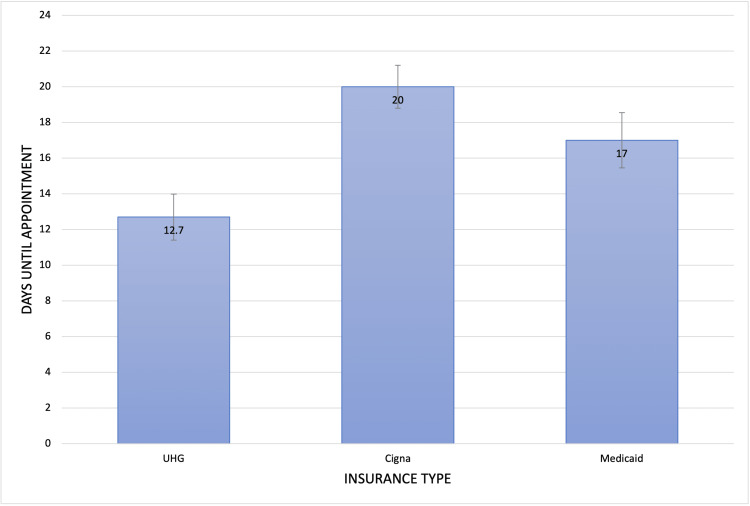
Average Number of Days Until Initial Prenatal Appointment This figure portrays the average number of days until the next available initial prenatal appointment, including standard error, for each insurance type studied.

Out of the total of 71 physicians who were successfully reached, 31 physicians accepted all three insurances, and 40 physicians did not accept at least one insurance. These 31 physicians were analyzed for the number of business days until an initial prenatal visit could be scheduled. The average number of days until the next available appointment was 12.7 (standard deviation (SD)= 7.2) for UnitedHealthcare, 20.0 (SD=6.7) for Cigna, and 17.0 (SD=8.6) for Medicaid (Figure 2, Table 2). There was a statistically significant trend between the type of insurance and the time to the earliest appointment (p=0.002). The average number of days until the next available appointment was compared between pairs of insurance types. There was a statistically significant difference in the number of days until the next available appointment when comparing UnitedHealthcare to Cigna (p<0.001) and when comparing UnitedHealthcare to Medicaid (p=0.037). However, there was no statistically significant difference between Cigna and Medicaid (p=0.193) (Table 3). Our study of Medicaid and two private insurances revealed that Medicaid is twice as likely to be rejected compared to both private insurances and has more than 1.3 times longer waiting periods before an initial appointment than UnitedHealthcare. 

**Table 2:** Analysis for Each Compared Insurance Type Based on Days to Appointment. This table includes the different types of analyses used when comparing insurance plans in this study, as well as the corresponding p-values for each analysis.

Type of Analysis	Insurance Plan Comparison	P- value
T- Test	Cigna vs. Medicaid	0.19327
	Cigna vs. UnitedHealthcare	0.00023879
	Medicaid vs. UnitedHealthcare	0.03734
ANOVA	Medicaid vs. Cigna vs. UnitedHealthcare	0.002152

**Table 3:** Days Until Initial Prenatal Appointment This table includes the total number of obstetricians who were contacted successfully for all three insurance types, the average number of days until the initial prenatal appointment for each insurance type, as well as the variance and standard deviation.

Insurance	Number of physicians contacted	Average number of days until appointment	Variance	Standard deviation
UnitedHealthcare	31	12.677	51.159	7.153
Cigna	31	19.548	44.589	6.678
Medicaid	31	16.968	74.499	8.631

## Discussion

The primary objective of our study was to assess the differences in access to obstetrical care between patients enrolled in private insurance versus Medicaid in South Florida. Medicaid in Florida provides coverage for adults with minor children whose income is less than or equal to 26% of the Federal Poverty Level (FPL) and pregnant women whose income is less than or equal to 196% of FPL for up to 12 months after childbirth [8]. We hypothesized that patients with private insurance plans would have greater acceptance by obstetricians and early access to initial prenatal appointments than those with government insurance plans. The three types of insurance analyzed in this study were Medicaid, representing government insurance, and two private insurance plans, UnitedHealthcare and Cigna. The study aimed to determine if women enrolled in Medicaid in South Florida have lower access to prenatal care than those enrolled in private insurance plans. Our study surveyed obstetricians to assess their availability and acceptance of various insurance types for scheduling initial prenatal appointments. To the best of our knowledge, this investigation represents the most extensive secret-shopper study of obstetricians in South Florida.

The study revealed a significant difference in acceptance rates between Medicaid and private insurance plans. Specifically, only 32 out of 71 providers (45.1%) accepted Medicaid, while almost all providers accepted private insurance plans. The acceptance rates for the private insurance plans were nearly identical, with 70 out of 71 providers (98.6%) accepting Cigna and 69 out of 71 (97.2%) accepting UnitedHealthcare. These results suggest that there may be disparities in access to obstetric care based on insurance type. These findings are consistent with a study published in 2019 by Hsiang et al., which examined the effect of insurance on scheduling appointments with primary care and specialty care providers [3]. The systematic review of 34 audit studies included 21,601 calls requesting Medicaid or private insurance appointments. The study found that patients with private insurance were 3.3 times more likely to successfully schedule appointments with specialty care providers (relative risk (RR) = 3.3, 95% confidence interval (CI) = 2.4-4.5) and 1.6 times more likely to schedule appointments with primary care physicians (RR = 1.6, 95% CI = 1.4-1.9) compared to patients with Medicaid [3]. These results further support our conclusion of disparities in healthcare access based on insurance type. One possible explanation for the low acceptance rate of Medicaid among obstetricians in South Florida is that it involves more complicated billing processes and offers lower reimbursement rates to physicians compared to private insurance plans [9]. This explanation is supported by a Gottlieb et al. (2018) study, which analyzed 37.2 million outpatient visits and 44.5 million submitted claims. The study found that the share of claims that were challenged and denied was higher for fee-for-service Medicaid (17.5% challenged, 21% denied) and managed care Medicaid (13.5% challenged, 9% denied) compared to private insurance plans, including UnitedHealthcare and Cigna (6% challenged, 4% denied) [9]. 

Our study also examined the average number of days until initial prenatal appointment for 31 obstetricians who accepted all three insurance plans. The results showed that UnitedHealthcare, a private insurance plan, had the shortest wait time of 12.7 days, while Cigna and Medicaid had wait times of 20.0 days and 17.0 days, respectively. There was a statistically significant trend between the type of insurance and the time to the earliest appointment (p=0.002). Although Cigna had the longest wait time among all insurance plans, there was no statistically significant difference between the number of days until the appointment between Cigna and Medicaid (p=0.193). These results raise the question of why Cigna, a private insurance company, had the longest wait time for the appointment despite its high acceptance rate. One suspected reason may be the differences in reimbursement rates between insurance plans. According to a study by Gottlieb published in 2018, out of the top five insurance companies, Cigna's challenged shares were 2.1 percentage points higher than fee-for-service Medicare (95% CI: −0.1, 4.3), while United Healthcare's challenged shares were 3.4 percentage points lower than fee-for-service Medicare (95% CI: 1.8, 5.0) [9]. Challenged shares are shares of authorized revenue given to providers for visits that were never paid. It is termed "challenged" as it is the amount challenged divided by the full negotiated amount. This suggests that Cigna's reimbursement rates for prenatal care may be lower than those of UnitedHealthcare and Medicaid, which could lead to longer wait times for prenatal appointments as providers may prioritize patients with higher reimbursement rates. However, further research would be needed to confirm this hypothesis.

The disparity between access to healthcare based on insurance type is a significant problem that can have negative effects on the health of pregnant patients and their fetuses. Early access to healthcare during pregnancy is crucial for identifying the risk of fetal aneuploidy and anatomic defects, as first-trimester aneuploidy screening allows the detection of more than 95% of affected fetuses with a false positive rate of < 3% as well as the overall well-being of the pregnant patient [10]. Specific screening tests in the first trimester include aneuploidy nuchal translucency and noninvasive prenatal testing, which test for Down syndrome, trisomy 13, and trisomy 18, and carrier screenings, including those for cystic fibrosis and spinal muscular atrophy. Our study investigates not only insurance acceptance rates but also the wait time for appointments. Timely prenatal care is crucial for identifying high-risk deliveries and referring patients to hospitals with higher levels of neonatology to decrease the risks of poor outcomes during and after deliveries [11-13]. If pregnant women do not receive appropriate and timely prenatal care, their health and the infant's health could be at risk [14]. A retrospective analysis by Partridge et al. in 2012 analyzed 28,729,765 US deliveries over eight years; it was determined that inadequate prenatal care, defined as prenatal care beginning after the fourth month of pregnancy or having less than 50% of the recommended number of visits, was associated with increased risk of prematurity, stillbirth, early neonatal death, late neonatal death, and infant death versus adequate prenatal care (p < 0.0001) [1]. The results of our study highlight a significant discrepancy in healthcare accessibility between individuals with private insurance and those with public insurance. This inequality and delay in receiving medical attention can lead to lasting effects on the health of both the patient and their family [15]. Access to basic healthcare during a natural part of life should not be dependent on one's insurance status. Our findings emphasize the need for improved access to healthcare for all individuals to ensure optimal health outcomes for pregnant patients and their fetuses. The results of this study should be considered in the future when making amendments or changes to these government plans to work on minimizing the discrepancy in access to obstetric care for women currently insured by Medicaid. 

Limitations 

The study had some limitations, which highlights the need for more research and data collection to strengthen the conclusions drawn. One limitation of this research study was the population sample; this population was limited to one state, Florida. Further research would benefit from expanding this geographical demographic to the southeast, east coast, or nationwide. Another limitation of this study was the use of a single database, HealthGrades.com. When selecting obstetricians to contact for potential appointments, we used a singular database and criteria. Unfortunately, this decreases the generalizability of our data compared to if we had cross-referenced obstetricians on multiple different databases and platforms to ensure we were getting a more accurate representation of the providers in Florida. Additionally, another limitation of our study was the need for more stratification within insurance companies. When calling obstetricians, often the assistant would inquire on which specific plan within Cigna the patient was a part of. It became evident that many obstetricians only accepted specific plans within an insurance company, with Cigna being the most questioned among the three insurance companies. In our data collection, we stated that our insurance plan was through our employment. However, more accurate results would have been if we stratified which plans were accepted versus denied. Finally, another limitation of the study was the fact that physicians were excluded if their contact information was incorrect and alternative phone numbers could not be found. This makes our data less representative of the actual acceptance rate in this population of obstetricians. 

## Conclusions

Literature has proven the importance of early onset of prenatal care for a fetus' gestational health. Consequently, if prenatal care is delayed for any reason, this could lead to dramatic intrapartum and postpartum consequences for both the mother and fetus. Through this cross-sectional study, the acceptance and time until the next available appointment varied based on what insurance a patient has. Coverage with a private insurance company allowed patients to be accepted by more obstetricians when compared to a public insurance company. Specifically, this study demonstrated that patients enrolled in Medicaid within Florida have significantly less access to prenatal care than those with private insurance. Our study of Medicaid and two private insurances revealed that Medicaid is twice as likely to be rejected compared to both private insurances and has more than 1.3 times longer waiting periods before visits than UnitedHealthcare. This evidence suggests that Medicaid plans may lead to decreased access to care, potentially increasing the risk of adverse outcomes related to inadequate prenatal care. Policymakers should consider this information when planning future Medicaid expansion. 
